# RETRACTION: A Meta‐Analysis Examining the Impact of Open Surgical Therapy versus Minimally Invasive Surgery on Wound Infection in Females With Cervical Cancer

**DOI:** 10.1111/iwj.70573

**Published:** 2025-04-18

## Abstract

**RETRACTION**: YunZ. , LiX. , ZhuD. , LiL. , and JiangS. , “A Meta‐Analysis Examining the Impact of Open Surgical Therapy versus Minimally Invasive Surgery on Wound Infection in Females With Cervical Cancer,” International Wound Journal 21, no. 4 (2024): e14535, 10.1111/iwj.14535.38169097 PMC10961045

The above article, published online on 02 January 2024, in Wiley Online Library (http://onlinelibrary.wiley.com/), has been retracted by agreement between the journal Editor in Chief, Professor Keith Harding; and John Wiley & Sons Ltd. Following an investigation by the publisher, all parties have concluded that this article was accepted solely on the basis of a compromised peer review process. In addition, the investigation found significant textual overlap in the methods sections between this article and other articles by different authors (Mao et al. 2023 [https://doi.org/10.1111/iwj.14203]; Wang et al. 2023 [https://doi.org/10.1111/iwj.14229]; and Zhang et al. 2023 [https://doi.org/10.1111/iwj.14179]). The editors have therefore decided to retract the article. The authors did not respond to our notice regarding the retraction.

**RETRACTION**: Z. Yun , X. Li , D. Zhu , L. Li , and S. Jiang , “A Meta‐Analysis Examining the Impact of Open Surgical Therapy versus Minimally Invasive Surgery on Wound Infection in Females With Cervical Cancer,” International Wound Journal 21, no. 4 (2024): e14535, 10.1111/iwj.14535.38169097 PMC10961045

The above article, published online on 02 January 2024, in Wiley Online Library (http://onlinelibrary.wiley.com/), has been retracted by agreement between the journal Editor in Chief, Professor Keith Harding; and John Wiley & Sons Ltd. Following an investigation by the publisher, all parties have concluded that this article was accepted solely on the basis of a compromised peer review process. In addition, the investigation found significant textual overlap in the methods sections between this article and other articles by different authors (Mao et al. 2023 [https://doi.org/10.1111/iwj.14203]; Wang et al. 2023 [https://doi.org/10.1111/iwj.14229]; and Zhang et al. 2023 [https://doi.org/10.1111/iwj.14179]). The editors have therefore decided to retract the article. The authors did not respond to our notice regarding the retraction.

